# Evaluation of “Dream Herb,”* Calea zacatechichi*, for Nephrotoxicity Using Human Kidney Proximal Tubule Cells

**DOI:** 10.1155/2016/9794570

**Published:** 2016-09-15

**Authors:** Miriam E. Mossoba, Thomas J. Flynn, Sanah Vohra, Paddy Wiesenfeld, Robert L. Sprando

**Affiliations:** Center for Food Safety and Applied Nutrition (CFSAN), Office of Applied Research and Safety Assessment (OARSA), Division of Toxicology (DOT), US Food and Drug Administration (US FDA), Neurotoxicology and In Vitro Toxicology Branch (NIVTB), 8301 Muirkirk Road, Laurel, MD 20708, USA

## Abstract

A recent surge in the use of dietary supplements, including herbal remedies, necessitates investigations into their safety profiles. “Dream herb,”* Calea zacatechichi*, has long been used in traditional folk medicine for a variety of purposes and is currently being marketed in the US for medicinal purposes, including diabetes treatment. Despite the inherent vulnerability of the renal system to xenobiotic toxicity, there is a lack of safety studies on the nephrotoxic potential of this herb. Additionally, the high frequency of diabetes-associated kidney disease makes safety screening of* C. zacatechichi* for safety especially important. We exposed human proximal tubule HK-2 cells to increasing doses of this herb alongside known toxicant and protectant control compounds to examine potential toxicity effects of *C. zacatechichi* relative to control compounds. We evaluated both cellular and mitochondrial functional changes related to toxicity of this dietary supplement and found that even at low doses evidence of cellular toxicity was significant. Moreover, these findings correlated with significantly elevated levels of nephrotoxicity biomarkers, lending further support for the need to further scrutinize the safety of this herbal dietary supplement.

## 1. Introduction

*Calea zacatechichi* (also called* Calea ternifolia* or “Dream Herb”) is a flowering plant native to Central America and has a long tradition of use as a medicinal plant in indigenous cultures [1]. Exposure through inhalation (smoking) or ingestion (as tea) is primarily used to temporarily intensify lucid dreaming. It is also widely consumed to treat problems associated with the gastrointestinal and endocrine systems [2]. It has recently been marketed as a dietary supplement in the management of diabetes due to its ability to induce hypoglycemic effects [3–5] although its mechanism(s) of action remain unclear.

The oneirogenic and other biological effects of* C. zacatechichi* are attributed in part to their flavones and germacrolides components [6–10]. However, flavones represent a class of flavonoids that have been shown to carry cytotoxic effects in part through induction of cytochrome P450 enzyme expression [11–13]. In addition, germacrolides are part of the class of sesquiterpene lactones, which can also exhibit negative effects on both prokaryotic and mammalian cells [14]. The cytotoxicity of both flavonoids and sesquiterpene lactones has been exploited for use as therapy against cancer [15, 16].

Despite clear evidence that at least some of the biologically active components of* C. zacatechichi* have the potential to be cytotoxic, safety evaluations of whole forms of this herbal supplement are lacking, especially ones that focus on the kidney. The kidneys use a complex transport system to eliminate unwanted chemicals, regulate blood pressure and glucose levels, and maintain a balanced pH [17]. However, as the glomerular filtrate passes through the tubular system, the reabsorption of water and electrolytes by the proximal tubule cells can progressively concentrate chemicals in the lumen that do not get reabsorbed. Unfortunately, the proximal tubules can become exposed to toxic concentrations of such chemicals, even when blood concentrations are relatively lower, leaving the kidneys vulnerable to injury [17]. In the case of* C. zacatechichi*, it is unknown whether any of its components can be nephrotoxic, but given that it is marketed to diabetics, any preexisting diabetic nephropathy marked by glomerular or proximal tubule damage [18–20] could induce further kidney damage. Therefore, we focused our research on screening for the potential nephrotoxicity of* C. zacatechichi* using an* in vitro* model of human proximal tubule cells. We chose the HK-2 cell line as our human proximal tubule model for its robust performance in many* in vitro* toxicology studies [21–25]. We compared the effects of exposing HK-2 cells to* C. zacatechichi* and two control compounds, a known renal toxicant (cisplatin) and a known renal protectant (valproic acid), and evaluated their dose-dependent effects on cytotoxicity, mitochondrial injury, and four kidney-specific biomarkers of toxicity [26–28]: (1) Kidney Injury Molecule-1 (KIM-1), (2) Albumin, (3) Cystatin C, and (4) *β*2-microglobulin (B2M). KIM-1 is expressed in tubular epithelial cells in response to injury. Albumin, Cystatin C, and B2M are indicators of impaired reabsorption by the proximal tubules. In this study, we demonstrate that* C. zacatechichi* is capable of inducing both cellular and organellar toxicity in proximal tubule cells.

## 2. Materials and Methods

### 2.1. Characterization of* Calea zacatechichi* Extract

Voucher samples of* C. zacatechichi* deposited at the University of Mississippi, National Center for Natural Products Research (NCNPR) (NCNPR #2443), were authenticated using macroscopy and microscopy methods by an NCNPR botanist. A methanol-extract of* C. zacatechichi* was provided in lyophilized form by NCNPR and was stored in the dark at 4°C in a vacuum chamber. Dried extract of* C. zacatechichi* was analyzed by LC/QTof as described previously [29]. Compounds were putatively identified by matching exact mass of analytes with components of* C. zacatechichi* reported in the literature [8, 30–33].

### 2.2. Cell Culture and Treatments

HK-2 cells were grown, maintained, and treated in a manner similar to that described previously [29]. Stock treatment solutions of* C. zacatechichi*, nephrotoxicant (positive control) cis-diamineplatinum(II) dichloride (cisplatin) (Sigma-Aldrich, St. Louis, MO), and nephroprotectant (negative control) valproic acid (Sigma-Aldrich) were made by weighing out their powders, dissolving them in DMSO, and diluting this mixture with media for a final DMSO stock solution of 0.4% or less. Cells were incubated overnight and treated in triplicate for 24 hours at the dose range of 0–1000 *μ*g/mL.

### 2.3. Cytotoxicity Assay

Treatment-related cytotoxicity was determined using the established CellTiter-Glo Cell Viability Assay (Promega, Madison, WI) following the manufacturer's recommendations. The premise of this luminescent assay is that ATP production is directly proportional to cell viability, as ATP is central to energy required for vital cellular processes. Treated cells in black-wall, clear bottom 96-well plates were equilibrated to room temperature for 30 minutes, during which time water in the outer wells was replaced with approximately 100 uL of treatment or media only controls. Following that, an equal volume of CellTiter-Glo working solution was added to each well. Plates were placed on an orbital shaker for 2 minutes to induce cell lysis and then incubated for an additional 10 minutes before being read on an OMG Fluorostar plate reader (BMG LABTECH, Ortenberg, Germany) to measure the levels of luminescence emitted from each well.

### 2.4. Reactive Oxygen Species Assay

Quantification of reactive oxygen species (ROS) was determined using Promega's ROS-Glo H_2_O_2_ luminescence-based detection system and data were normalized to cell viability. Following 24 hrs of direct exposure to* C. zacatechichi,* cells were incubated with H_2_O_2_ substrate and detection reagent, as recommended in the manufacturer's instructions. Luminescence was read on an OMG Fluorostar plate reader.

### 2.5. Mitochondrial Membrane Potential Assay

Changes in mitochondrial membrane potential (MMP) were evaluated using the ratiometric dye JC-10 (Enzo, Farmingdale, NY). HK-2 cells that were directly exposed to* C. zacatechichi* were stained with 20 uM JC-10 (final concentration) for 2 hours, washed, and then read by plate reader (OMG Fluorostar). Excitation was set at 485 nm and emission at 520 and 590 nm was measured. We also verified that extract or media alone did not produce significant emission signals.

### 2.6. Nephrotoxicity Biomarker Assays

Culture supernatants from cells treated for 24 hours with* C. zacatechichi*, cisplatin, and valproic acid at doses of 333 and 111 *μ*g/mL were evaluated for levels of biomarkers of kidney toxicity: Kidney Injury-1 (KIM-1), Albumin, Cystatin C, and beta-2-microglobulin (B2M) using the Human Kidney Toxicity kits (Bio-Rad, Hercules, CA). Following the manufacturer's protocol, plates were blocked, washed, and incubated with samples, standard solutions detection antibodies, before being given a final wash. Plates were read using a Luminex 200 instrument (Bio-Rad). Biomarker expression levels were normalized to cell viability.

### 2.7. Statistics

Microsoft Excel and Prism (GraphPad, San Diego, CA) were used for calculations and analyses of all data collected. Student's *t*-tests or 2-way ANOVAs were used to determine whether dose-matched treatment effects were statistically significant at *P* values less than 0.01 or 0.001 as indicated.

## 3. Results

### 3.1. Characterization of* Calea zacatechichi* Extract

**Figure 1:**
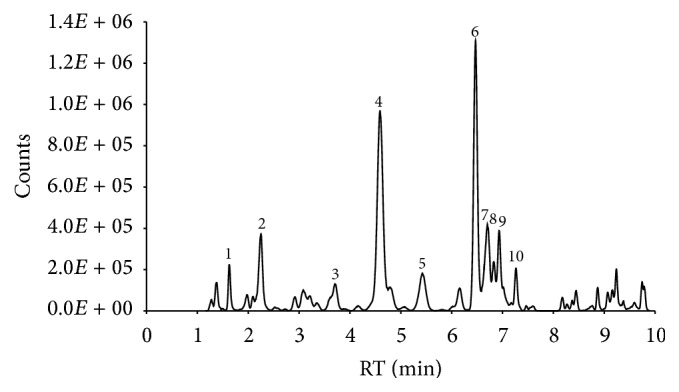
Extracted total compound chromatogram from chemical characterization of the* C. zacatechichi* extract by LC-high resolution mass spectroscopy. Putative compound identification was made by matching exact mass with that of known components of* C. zacatechichi* [8, 30–33]. (1) Ciliarin, (2) zexbrevin, (3) sesquiterpene lactone, (4) calein D, (5) 1-*β*-acetoxyzacatechinolide, (6) calein A, (7) 1-oxo-zacatechinolide, (8) calealactone E, (9) calealactone, and (10) acetoxycaleculatolide.

LC-high resolution MS found 231 total molecular features in the* C. zacatechichi* extract. Of these, 24 features had exact mass consistent with that of reported components of* C. zacatechichi* (Figure 1). The major components based on peak volume, calein A, ciliarin, acacetin, and calealactone C, accounted for about 50% of the known compounds and 8% of the total compounds [8, 30–33].

### 3.2. *C. zacatechichi* Strongly Inhibits HK-2 Cell Viability

**Figure 2:**
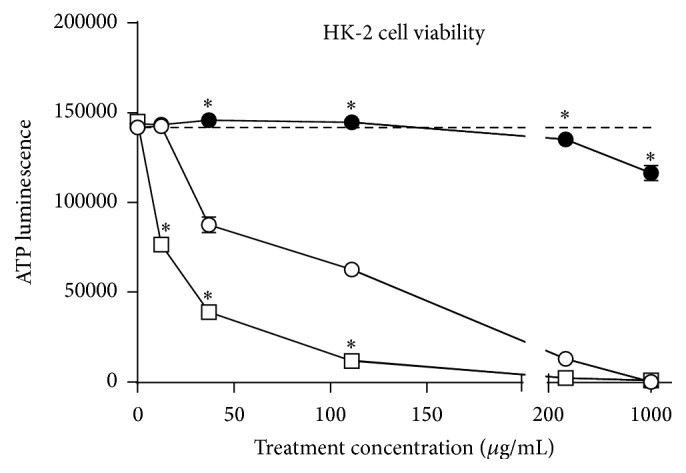
*C. zacatechichi* significantly decreases cell viability in a dose-dependent manner. HK-2 cells were treated with* C. zacatechichi* (open circles), cisplatin (open squares), or valproic acid (filled circles) at mean average concentrations (± SEM) ranging from 0 to 1000 *μ*g/mL and cell viability was quantitatively assayed by ATP luminescence 24 hours after treatment. Dashed line indicates “no treatment” baseline ATP levels. *∗*, cisplatin or valproic acid versus* C. zacatechichi*, *P* < 0.001.

To investigate the nephrotoxicity of* C. zacatechichi*, we performed an ATP-based cell viability assay on HK-2 cells treated with a 6-dose concentration range from 0 to 1000 *μ*g/mL for 24 hours. For comparison, we also treated cells for 24 hours with dose-matched concentrations of the known nephrotoxic compound, cisplatin, and the known nephroprotectant, valproic acid. We found that cisplatin induced a significant reduction in cell viability starting at the ~12 *μ*g/mL dose tested (*P* < 0.001) and caused complete cell death at the maximum dose tested (Figure 2). Similarly, significant cytotoxicity of* C. zacatechichi* was detected starting at 37.0 *μ*g/mL (*P* < 0.001) and still achieved complete cell death by 1000 *μ*g/mL. For the range of doses tested, the cytotoxic effect of* C. zacatechichi* was directly proportional to the treatment dose and we calculated its lethal concentration 50 (LC_50_) value to be 91.7 *μ*g/mL, compared to 13.3 *μ*g/mL for cisplatin. By contrast, valproic acid successfully maintained cell viability across the range of tested doses, except for the maximum dose of 1000 *μ*g/mL, where cell viability dropped only slightly, as shown in Figure 2. As expected from a nephroprotectant, the calculated LC_50_ value of valproic acid is quite high at 3866 *μ*g/mL, given the plateau shape of its cell viability curve.

### 3.3. Mitochondrial Toxicity Increases Proportionately with Higher Exposure to* C. zacatechichi*

**Figure 3:**
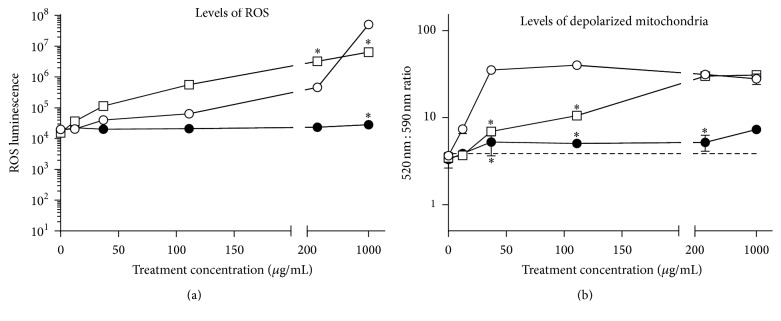
Cellular stress induced by* C. zacatechichi* is indicated by a surge in ROS and a rapid shift toward MMP loss. HK-2 cells treated for 24 hours with* C. zacatechichi* (open circles), cisplatin (open squares), or valproic acid (filled circles) were assayed for cellular levels of (a) normalized mean average ROS levels (± SEM) as well as (b) changes in the relative amounts of mitochondria that undergo loss versus maintenance of membrane potential, calculated as a mean average (± SEM) ratio of fluorescence emission at 520 versus 590 nm. *∗*, cisplatin or valproic acid versus* C. zacatechichi*, *P* < 0.001.

To begin studying early events of cellular toxicity, we evaluated how the mitochondria of HK-2 cells were affected by 24-hour treatments with* C. zacatechichi* relative to treatments with cisplatin or valproic acid. We first measured the levels of ROS produced in treated cells to indicate the levels of oxidative stress that was created in the intracellular environment of HK-2 cells. Using a luminescence assay of ROS detection, we found that* C. zacatechichi* treatment led to increasingly higher levels of ROS production in a manner that was directly proportional to the increasing treatment dose (Figure 3(a)). ROS production from cells treated with up to 333 *μ*g/mL of* C. zacatechichi* was intermediary between those from the positive- and negative-control treated cells. At the 1000 *μ*g/mL dose, however,* C. zacatechichi* induced ROS levels that surpassed those in cisplatin-treated cells (*P* < 0.001), as shown in Figure 3(a).

To gain a better understanding of (1) whether the elevated levels of ROS production actually correlated with mitochondrial injury and (2) to what extent injury took place, we performed a ratiometric assay using JC-10 dye to compare the relative levels of damaged and healthy mitochondria in treated HK-2 cells. Compared to the baseline ratio value of about 5, treatment with* C. zacatechichi* led to a uniquely sharp increase in the ratio of damaged to healthy mitochondria starting from the 12.3 *μ*g/mL testing dose and achieved a maximum ratio value of about 50 when the treatment dose was increased to just 37.0 *μ*g/mL (Figure 3(b)). This maximal relative level of mitochondrial damage was statistically significant (*P* < 0.001) and was well sustained for the remaining higher treatment doses of* C. zacatechichi*. By contrast, cisplatin induced mitochondrial damage at a much slower rate to achieve a ratio value of ~50 at 333 *μ*g/mL. As expected, the mitochondrial injurious effects from valproic acid were minimal over the spectrum of treatment doses.

### 3.4. Proximal Tubule Cell Function Is Significantly Compromised by* C. zacatechichi*

**Figure 4:**
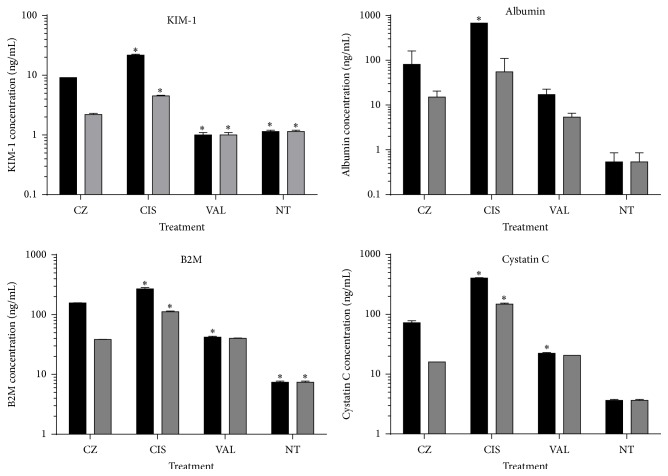
Biomarker signature of* C. zacatechichi*-associated nephrotoxicity. HK-2 cells were treated with* C. zacatechichi *(CZ), cisplatin (CIS), or valproic acid (VAL) or left untreated (NT). Treatment concentrations were either 333 *μ*g/mL (black bars) or 111 *μ*g/mL (gray bars). At 24 hours after treatment, HK-2 cell culture supernatants were harvested and assayed by Bio-Plex assay for average levels of KIM-1, Albumin, Cystatin C, and B2M (± SEM). Biomarker expression levels were normalized to cell viability. *∗*, cisplatin or valproic acid versus* C. zacatechichi*, *P* < 0.01.

To address whether renal cell function would become compromised after treatment with* C. zacatechichi*, we evaluated cellular biomarkers that are strong indicators of nephrotoxicity [26–28]. We used a sensitive multiplex approach to simultaneously detect differences in the levels of four FDA-qualified biomarkers: KIM-1, Albumin, Cystatin C, and B2M. We quantitated the concentrations of these analytes in the culture supernatants of HK-2 cells exposed to 111 or 333 *μ*g/mL of* C. zacatechichi*, cisplatin, valproic acid, or untreated media for 24 hours (Figure 4). In agreement with our findings of cellular and mitochondrial toxicity, we found significantly elevated levels for nearly all of these markers (*P* < 0.01) in culture supernatants of HK-2 cells treated with* C. zacatechichi* compared to those treated with valproic acid or left untreated. The extent of biomarker elevation induced by* C. zacatechichi* never exceeded that of cisplatin. This trend was also observed at the lower treatment dose of 111 *μ*g/mL, even though the actual concentrations of each biomarker were typically 10-fold less than in high-dose treatments, as shown in Figure 4.

## 4. Discussion

Although* C. zacatechichi* is not a controlled substance under United States federal law, it has been banned in the state of Louisiana as well as in Poland on the basis of its mind-altering effects [34, 35]. In our study, we used an* in vitro* human renal proximal tubule cell model to perform several assays that collectively evaluated the nephrotoxicity potential of* C. zacatechichi*. We used its alcohol extract to best model the tincture dietary supplements marketed in the United States. By comparing its toxicity profile to that of a highly toxic pure compound, cisplatin, and an innocuous pure compound, valproic acid, we established a stringent* in vitro* cell culture safety evaluation model system. Although we identified several of the chemical components of* C. zacatechichi*, we were focused on evaluating the toxicity of this herbal extract as a whole.* In vitro* testing not only provides a window into cell-specific effects [36] but also yields informative data on the mechanism(s) of toxicity [37]. We chose to use the human renal proximal tubule epithelial cell line, HK-2, because the proximal tubule plays a critical role in controlling the clearance and reabsorption processes of xenobiotics and their metabolites [38, 39]. Proximal tubule epithelial cells encounter toxicants that are filtered and are an important component of overall nephrotoxicity that can lead to both acute and chronic kidney damage [40]. The HK-2 cell line is an appropriate choice for establishing a renal cell toxicology profile on* C. zacatechichi* for two main reasons. First, HK-2 cells are human derived and thus, data generated from this cell line are not confounded by differences between human and other species. Second, HK-2 cells closely recapitulate many aspects of the morphological and metabolic phenotype of proximal tubule cells* in vivo* [23, 41]. In our evaluation of cytotoxicity, we found striking similarities between our tested herbal extract and cisplatin at single dose treatment concentrations as low as approximately 37 *μ*g/mL in the form of short-term exposure. Moreover, it appeared that the mechanism of action of* C. zacatechichi*'s active ingredients or renal-derived metabolites resembled those of the highly injurious cisplatin; elevated ROS levels and a severe loss of mitochondrial membrane potential were hallmarks of nephrotoxicity shared by these two substances. By contrast, valproic acid showed little or no toxicity potential until the highest dose of 1000 *μ*g/mL was tested. The relatively high level of toxicity that was induced by* C. zacatechichi* within the 24 hours of direct exposure to HK-2 cells is of concern. However, since no data exist on the serum concentrations of* C. zacatechichi*'s active components, it is unclear how our chosen treatment doses compare to what kidney cells* in vivo* would be exposed to, especially postmetabolism by the gut and liver.

Although further studies would be needed to elucidate a more detailed mechanistic analysis of* C. zacatechichi*'s modes of action, we have found additional evidence of its potential to cause significant renal cell damage. Specifically, our panel of indicators of kidney injury showed that not only were these biomarkers elevated, but also the intensity of their elevation approached that measured in our assays following cisplatin treatment. The biomarkers we selected included those that have been qualified by the FDA to serve as official biomarkers of nephrotoxicity. They have gained attention recently as they have been shown to be solid correlates of* in vivo* nephrotoxicity [42]. Overall, our findings indicated that the cellular toxicity of* C. zacatechichi* was capable of producing elevations in all four biomarkers at the high treatment dose, but to a lesser extent than cisplatin.

Other toxicology studies on* C. zacatechichi* using* in vivo* model systems have not specifically evaluated nephrotoxicity endpoints but have still demonstrated its potential to have a range of side effects. In a rat model, for example, extracts of this herb were reported to inhibit edema and neutrophil migration [43]. In a feline model, it caused ataxia, vomiting, and unusual electroencephalogram (EEG) recordings [44]. In human volunteers, it resulted in significant increases in respiratory rates and decreases in reaction times [44].

In support of the idea that* C. zacatechichi* has the potential to cause cell injury, other groups have shown that extracts of this herb or its purified components can exert inhibitory effects on cells using* in vitro* model systems. For example, a recent toxicology study has shown that* C. zacatechichi* can inhibit the transcription factor NF-kappaB, which is critical to regulating cellular inflammation and other functions [45, 46]. A further understanding of* C. zacatechichi*'s mechanism(s) of action may be extrapolated from studies on other members of the* Calea* genus. For example,* C. platylepis*,* C. uniflora*, and* C. serrata* have been shown to possess potent antimicrobial, antifungal, and acaricidal activities, respectively [47–49]. In addition,* C. pinnatifida* was shown to have cytotoxic effects against a wide variety of human cell lines derived from a variety of organ systems, including kidney [50]. Moreover, studies on germacrolides, which are common components of most herbs in the* Calea* genus, including* C. zacatechichi*, have demonstrated the potential for antileishmania effects [8], inhibition of cellular differentiation [51], and cytotoxicity against human leukemia cells [52, 53].

Taken together,* C. zacatechichi* or its components may pose unwanted health effects, especially if long-term daily doses are taken to control hyperglycemia. Our* in vitro* HK-2 proximal tubule cell model depicted potentially nephrotoxic features of this herb at both the cellular and organellar levels. It would be pertinent to next perform an* in vivo* investigation of its systemic and organ-specific effects, including those on the other parts of the kidney.

## Additional Points

*Highlights.* (i)* In vitro* exposure of human kidney cells to* Calea zacatechichi* is cytotoxic.

(ii) Mechanism of cytotoxicity may involve ROS production and mitochondrial injury.

(iii) Biomarkers of nephrotoxicity are elevated following* in vitro* exposure to* Calea zacatechichi*.

## Abbreviations

ROS: Reactive oxygen species
MMP: Mitochondrial membrane potential
KIM-1: Kidney Injury Molecule-1
B2M: beta-2-microglobulin.
